# Litter quality drives the differentiation of microbial communities in the litter horizon across an alpine treeline ecotone in the eastern Tibetan Plateau

**DOI:** 10.1038/s41598-018-28150-1

**Published:** 2018-07-03

**Authors:** Haifeng Zheng, Yamei Chen, Yang Liu, Jian Zhang, Wanqing Yang, Lin Yang, Hongjie Li, Lifeng Wang, Fuzhong Wu, Li Guo

**Affiliations:** 1 Key Laboratory of Ecological Forestry Engineering of Sichuan Province, Institute of Ecology and Forestry, Sichuan Agricultural University, Chengdu, 611130 China; 2 College of Landscape Architecture, Sichuan Agricultural University, Chengdu, 611130 China

## Abstract

Cellulose and lignin are the main polymeric components of the forest litter horizon. We monitored microbial community composition using phospholipid fatty acid (PLFA) analysis and investigated the ligninolytic and cellulolytic enzyme activities of the litter horizon across an alpine treeline ecotone in the eastern Tibetan Plateau. The activities of ligninolytic and cellulolytic enzymes and the biomass of microbial PLFAs were higher in the initial stage of litter decomposition than in the latter stage in the three vegetation types (coniferous forest, alpine shrubland and alpine meadow). Soil microbial community structure varied significantly over the course of litter decomposition in the three vegetation types. Furthermore, the BIOENV procedure revealed that the carbon to nitrogen (C:N) ratio, carbon to phosphorus (C:P) ratio and moisture content (MC) were the most important determinants of microbial community structure in the initial stage of litter decomposition, whereas pH and the lignin concentration were the major factors influencing the microbial community structure in the later stage of litter decomposition. These findings indicate that litter quality drives the differentiation of microbial communities in the litter horizon across an alpine treeline ecotone in the eastern Tibetan Plateau.

## Introduction

The decomposition of plant litter involves a complex set of processes that include chemical, physical, and biological agents acting upon a wide variety of organic substrates. Based on the degree of decomposition, the litter horizon can typically be divided into a fresh litter (L) layer, a fermentation (F) layer and a humus (H) layer^1–3^. These three layers largely represent different degrees of decomposition of organic matter. The L layer is the upper layer and is formed by recognizable plant and soil animal remains. Below this layer is a layer that typically consists of a mixture of organic matter in different stages of decomposition, known as the F layer. The third layer is the H layer, which largely consists of humified material with little or no visible plant structure^1,2^. However, in some cases, the F layer is thin or absent and difficult to distinguish from the L layer. Therefore, the litter horizon can also be divided into a fresh litter and fermentation (LF) layer and a humus (H) layer^4^, which represent the initial and later stages of litter decomposition, respectively^4,5^. The LF layer is the upper 2 cm layer that contains easily recognizable plant remains that show some discoloration^4,6^. The 2 to 5 cm layer comprises humified material without recognizable plant structures except for stems and coarse root remains, and it is referred to as the H layer^4^. Some nutrients and low-molecular-weight compounds, notably sugars, are readily lost from the litter through dissolution and leaching in the initial stages of litter decomposition and due to the action of rapidly growing microorganisms. In contrast, larger macromolecules, such as cellulose, hemicelluloses, and lignin, are degraded more slowly in the later stages of litter decomposition^7^. During soil transformation, the litter horizon is formed from both lignocellulose and the structural components of microbial decomposers^8^. Moreover, the degradation of lignocellulose is a critical component of the decomposition of the fraction of global carbon (C) in the soil, which is the largest C pool on Earth.

Microorganisms, such as white rot, brown rot, and soft rot, are the primary decomposers in forest soil systems and the main producers of enzymes that decompose lignin and cellulose^1,9^, the two most abundant phytochemicals in soils. Thus, microorganisms are the most important players in litter decomposition^10,11^. The microbial products of decomposition become the main precursors of stable soil organic matter by promoting aggregation and by forming strong chemical bonds with the mineral soil matrix^12^. Ligninases and cellulases are important extracellular C-acquiring enzymes involved in litter decomposition. The lignin decomposition system consists of three principle ligninases: laccase, manganese peroxidase (MnP) and lignin peroxidase (LiP)^13^. Cellulose-degrading microorganisms secrete numerous cellulolytic enzymes (mainly cellobiohydrolase, endo-1,4-β-D-glucanase (EG) and β-glucosidase (BG)), which act synergistically to completely degrade lignocellulosic biomass^14,15^.

According to previous studies, litter substrate quality varies with the stage of litter decomposition^1,16^. However, little is known regarding the determinants of microbial communities and the differentiation of the relationships between litter chemical properties and the structure of the microbial community in the different stages of litter decomposition. The Microbial Efficiency-Matrix Stabilization (MEMS) framework suggests that labile plant constituents are utilized more efficiently by microbes during the initial stages of litter decomposition^12^. Due to the differences among the different stages of litter decomposition in chemical properties and available carbon for microorganisms, we hypothesized that lignocellulolytic enzyme activity and microbial community structure are constantly changing during the different stages of litter decomposition.

The alpine treeline ecotone is the zone that extends from closed subalpine forest (timberline) to the upper boundary of tree distribution. This ecotone does not typically occur as an abrupt line; rather, it typically appears as a patchy transition that consists of several intermediate vegetation zones and components, such as dark coniferous forest, alpine shrubland and alpine meadow^17^. An alpine climate and low land-surface temperatures (with an annual average temperature of 6 to 12 °C) result in a low rate of litter decomposition in the alpine treeline ecotone. Previously, we studied the litter decomposition rate and the release of carbon and nutrients during litter decomposition^17^ and the mass loss and lignocellulolytic enzyme activities of residues and leaf litter in a coniferous forest and timberline^5^. However, we did not investigate microbial community structure during litter decomposition. Therefore, in the present study, we monitored microbial community composition using phospholipid fatty acid (PLFA) analysis and assessed the ligninolytic and cellulolytic enzyme activities of the litter horizon across an alpine treeline ecotone in the eastern Tibetan Plateau. Our objectives were to answer two questions: (1) How do lignocellulolytic enzyme activities, microbial PLFA biomasses and community structure differ between the two litter decomposition stages for the three vegetation types? (2) How do the relationships between litter physicochemical properties (e.g., soil organic carbon (SOC), total nitrogen (TN) and total phosphorus (TP)) and microbial community structure differ between the two litter decomposition stages?

## Results

### Litter physicochemical properties

The SOC, lignin and cellulose concentrations and the C:N, C:P, and N:P ratios of the LF layer were higher than those of the H layer for all three vegetation types (coniferous forest, alpine shrubland and alpine meadow) (all *p* < 0.05, Table 1), and TP was lower in the LF layer than in the H layer for all three vegetation types (all *p* < 0.05, Table 1). There was no significant difference in TN between the LF and H layers for any of the three vegetation types (Table 1). Moisture content and pH were lower in the LF layer than in the H layer in alpine meadow (*p* < 0.05, Table 1). pH was higher in the LF layer than in the H layer in coniferous forest (*p* < 0.05, Table 1).

**Table 1:** Chemical properties of the litter layers.

Variable	Coniferous forest		Alpine shrubland		Alpine meadow	
	LF layer	H layer	LF layer	H layer	LF layer	H layer
SOC (g kg^–1^)	330 ± 40.9a	201.8 ± 51.5b	284.5 ± 70.1a	210.2 ± 81.7b	415.7 ± 42.3a	115.4 ± 26.0b
TN (g kg^–1^)	9.4 ± 1.3a	8.5 ± 1.0a	9.4 ± 2.1a	8.8 ± 0.8a	7.4 ± 0.7a	7.1 ± 1.3a
TP (g kg^–1^)	2.3 ± 0.6b	2.9 ± 0.5a	2.1 ± 0.5b	2.4 ± 0.4a	1.9 ± 0.7b	2.4 ± 0.3a
C:N	35.5 ± 5.4a	23.5 ± 4.5b	30.4 ± 4.9a	23.7 ± 8.5b	57.0 ± 8.9a	16.4 ± 2.5b
C:P	152.8 ± 35a	72.6 ± 22.8b	141.5 ± 38a	86.8 ± 31.5b	245.8 ± 76.7a	49 ± 11.4b
N:P	4.3 ± 0.8a	3.1 ± 0.7b	4.7 ± 1.4a	3.7 ± 0.5b	4.4 ± 1.6a	3.0 ± 0.5b
MC (%)	54.0 ± 15.2a	48.9 ± 13.3a	60 ± 8.3a	60.4 ± 5a	16.4 ± 6.7b	46.2 ± 6.3a
pH	5.5 ± 0.2a	4.8 ± 0.2b	5.7 ± 0.2a	5.5 ± 0.3a	5.7 ± 0.1b	6.0 ± 0.1a
Cellulose (%)	34.8 ± 5.9a	16.3 ± 6.3b	28.5 ± 11.4a	16.1 ± 6b	37.0 ± 4.6a	7.9 ± 3.1b
Lignin (%)	25.5 ± 3.5a	14.8 ± 6.1b	17.1 ± 6.4a	14.3 ± 3.4a	12.2 ± 5a	6.2 ± 2.9b

The results are the means ± standard errors of 15 replicates. Lowercase letters indicate significant differences (*p* < 0.05) between different layers within the same vegetation type as identified by the Mann-Whitney U test. SOC, soil organic carbon; TN, total nitrogen; TP, total phosphorus; MC, moisture content.

### Lignocellulolytic enzyme activity

**Figure 1:**
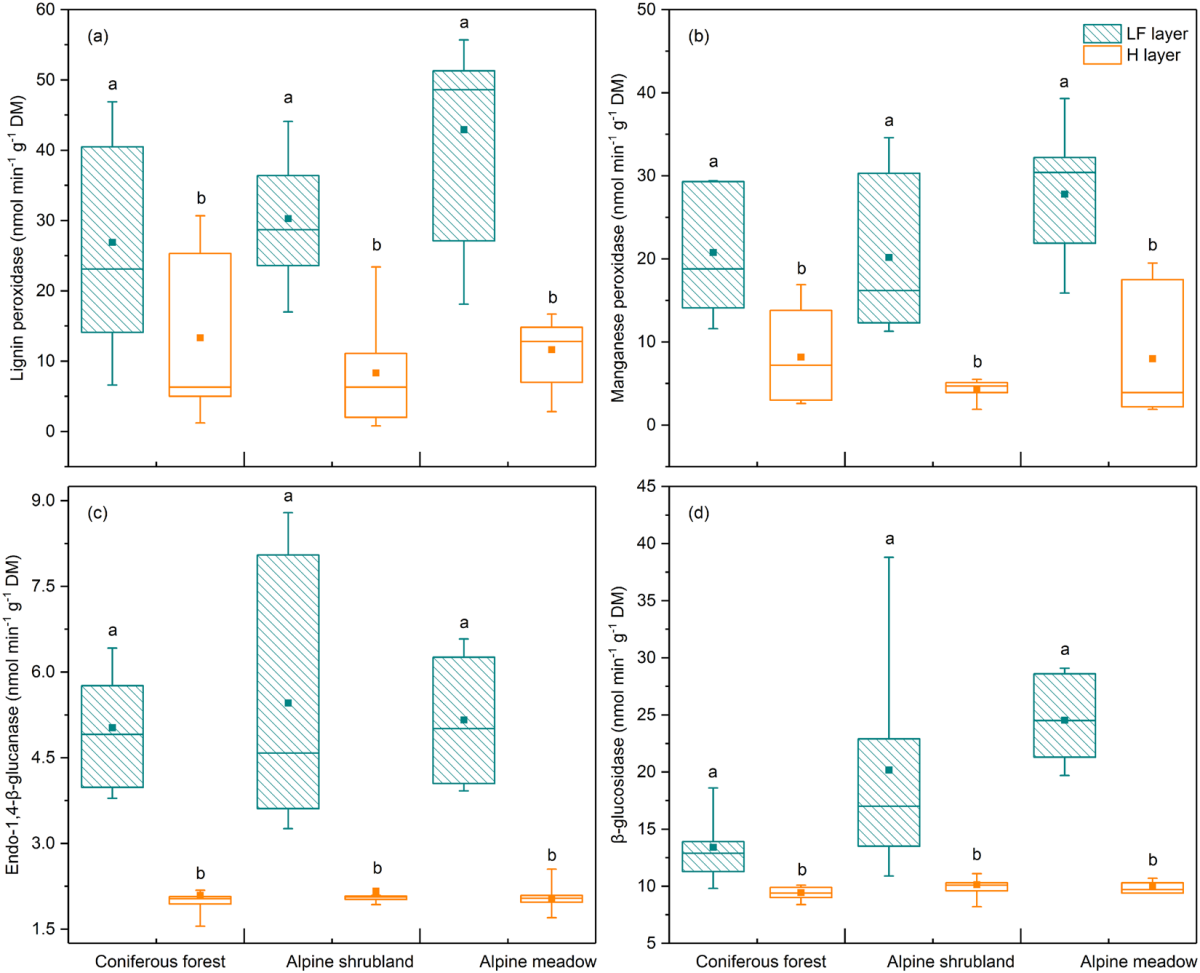
Enzymatic activities in the LF and H layers in coniferous forest, alpine shrubland and alpine meadow. The boxes represent the range of the first and third quartiles; the horizontal lines in the boxes represent the median; points within each boxplot represent the means; the upper and lower bounds of the bars reflect the 90th and 10th percentiles, respectively. Bars with different lowercase letters indicate significant differences (*p* < 0.05) between the LF and H layers within the same vegetation type as identified by the Mann-Whitney U test.

The activities of LiP, MnP, EG, and BG were significantly higher in the LF layer than in the H layer in all three vegetation types (all *p* < 0.05, Fig. 1). The Pearson correlation analysis showed that the activities of LiP, MnP, EG, and BG were positively correlated with SOC; the C:N, C:P and N:P ratios; and cellulose concentrations (all *p* < 0.01, Table 2). The activities of LiP, MnP, EG, and BG were negatively correlated with TP and MC (except MnP with TP, *p* < 0.05, and EG with MC, *p* = 0.206; all *p* < 0.01, Table 2).

**Table 2:** Pearson correlation coefficients of enzyme activities and chemical properties (n = 90).

Enzyme	SOC	TN	TP	C:N	C:P	N:P	MC	pH	Cel	Lig
LiP	0.577^**^	−0.040	−0.420^**^	0.627^**^	0.682^**^	0.425^**^	−0.450^**^	0.135	0.581^**^	0.091
MnP	0.663^**^	0.116	−0.265^*^	0.632^**^	0.639^**^	0.371^**^	−0.372^**^	0.147	0.673^**^	0.242^*^
EG	0.617^**^	0.165	−0.330^**^	0.556^**^	0.608^**^	0.475^**^	−0.135	0.143	0.676^**^	0.340^**^
BG	0.617^**^	0.071	−0.436^**^	0.624^**^	0.691^**^	0.499^**^	−0.457^**^	0.250^*^	0.536^**^	0.032

Values are Pearson correlation coefficients. “*” and “**” indicate significance at the 0.05 and 0.01 levels, respectively. Enzyme abbreviations are defined in Table 5. SOC, soil organic carbon; TN, total nitrogen; TP, total phosphorus; MC, moisture content; Cel, cellulose concentration; Lig, lignin concentration

### The biomass of microbial PLFAs and microbial community structure

**Figure 2:**
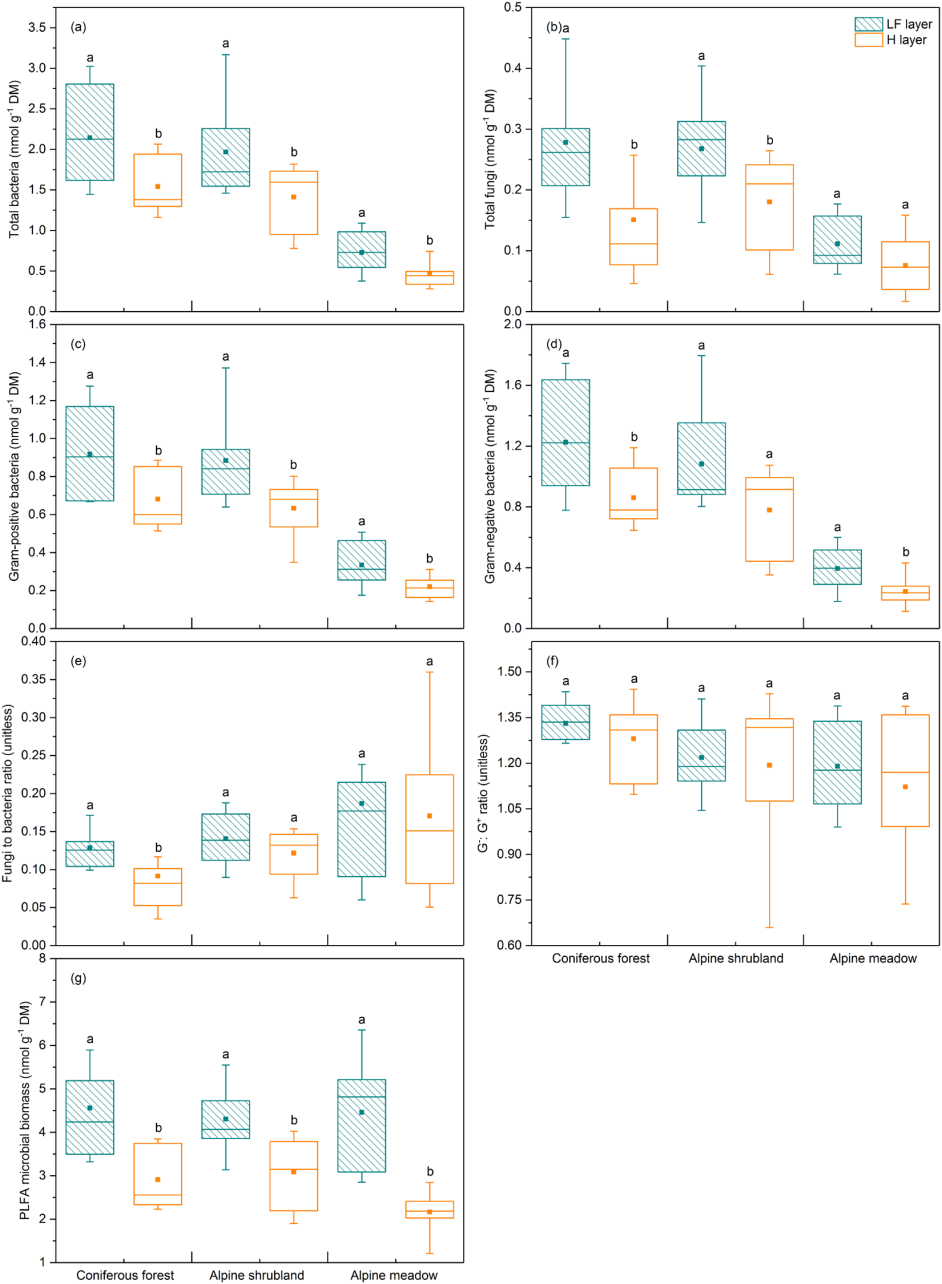
The concentrations of microbial PLFAs and ratios of biomarkers in the LF and H layers in coniferous forest, alpine shrubland and alpine meadow. The boxes represent the range of the first and third quartiles; the horizontal lines in the boxes represent the median; points within each boxplot represent the means; the upper and lower bounds of the bars reflect the 90th and 10th percentiles, respectively. Bars with different lowercase letters indicate significant differences (*p* < 0.05) between the LF and H layers within the same vegetation type as identified by the Mann-Whitney U test. G^−^:G^+^, ratio of gram-negative bacteria to gram-positive bacteria.

The PLFA contents of TB (total bacteria), TF (total fungi), G^+^ (gram-positive bacteria), G^−^ (gram-negative bacteria) and MB (microbial biomass) of the LF layer were significantly higher than those of the H layer in coniferous forest (all *p* < 0.05, Fig. 2). The PLFA contents of TB, TF, G^+^ and MB were significantly higher in the LF layer than in the H layer in alpine shrubland (all *p* < 0.05, Fig. 2). The PLFA contents of TB, G^+^, G^−^ and MB were markedly higher in the LF layer than in the H layer in alpine meadow (all *p* < 0.05, Fig. 2). For the ratio of fungi to bacteria (F:B), a significant difference between the LF and H layers was observed only in coniferous forest (*p* < 0.05, Fig. 2e). No significant difference in the ratio of gram-negative to gram-positive bacteria (G^−^:G^+^) between the LF and H layers was observed for any of the three vegetation types (coniferous forest, alpine shrubland and alpine meadow) (Fig. 2f).

**Figure 3:**
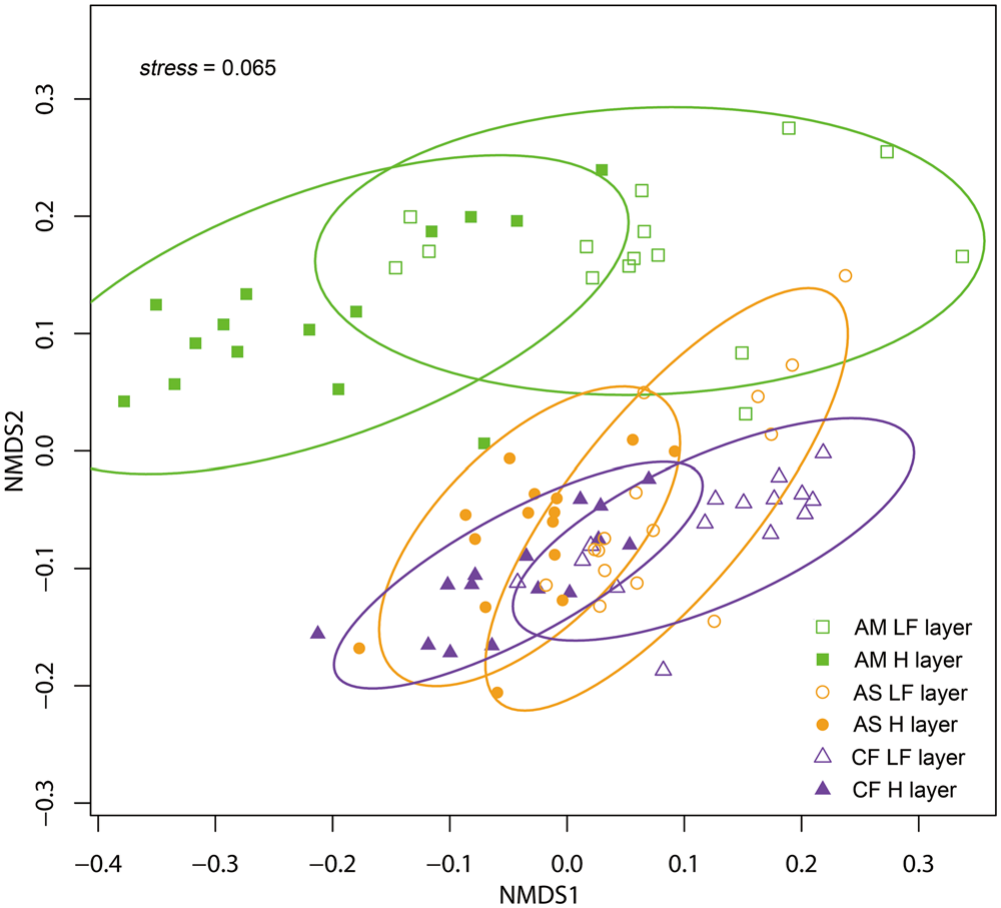
Bray-Curtis-based NMDS of the PLFAs in the LF and H layers in coniferous forest (CF), alpine shrubland (AS) and alpine meadow (AM).

The non-metric multidimensional scaling (NMDS) analysis clearly showed variation in the microbial community between the LF and H layers in all three vegetation types (stress = 0.065, Fig. 3). The significance of the patterns observed in NMDS was confirmed by PERMANOVA (all *p* < 0.01, Table 3).

**Table 3:** Results from the PERMANOVA. Microbial community Bray-Curtis dissimilarity was modeled as response variable.

Vegetation type	pair	*F*	*R* ^2^	*p*
Coniferous forest	LF layer vs. H layer	20.709	0.425	0.001
Alpine shrubland	LF layer vs H layer	9.507	0.253	0.001
Alpine meadow	LF layer vs H layer	19.111	0.406	0.001

### Correlations between microbial community structure, litter physicochemical properties and enzyme activities

**Figure 4:**
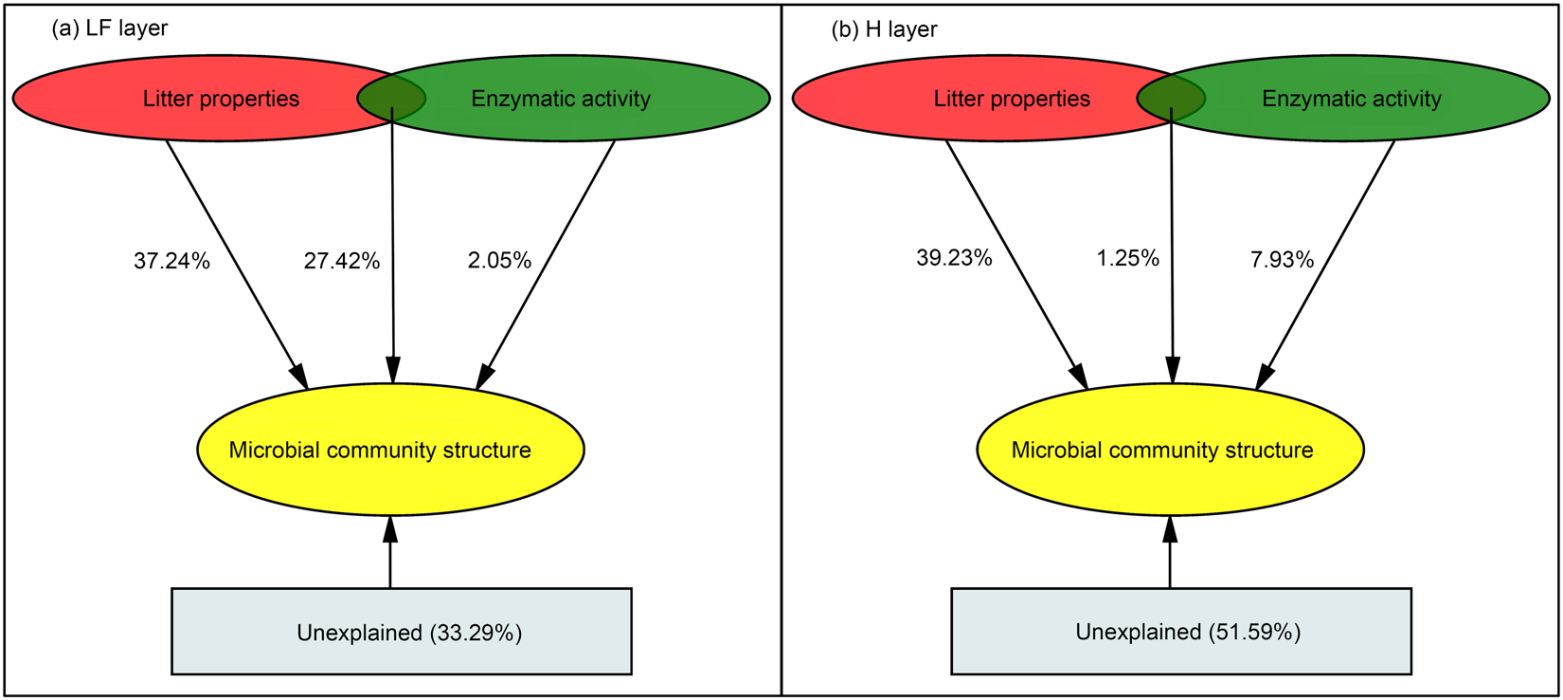
Variation partitioning analysis (VPA) of the effects of litter properties and enzymatic activity on microbial community structure in the LF (**a**) and H (**b**) layers. Overlapping areas represent the combined effects of litter properties and enzymatic activity.

Based on a variation partitioning analysis (VPA), microbial community structure in the LF and H layers was well explained by the litter physicochemical properties and enzyme activities. In the LF and H layers, 66.71% and 48.41% of the microbial community structure, respectively, could be explained mainly by physicochemical properties (37.24% in the LF layer and 39.23% in the H layer) (Fig. 4). Only 2.03% and 7.93% of the microbial community structure in the LF layer and H layer, respectively, could be explained by enzyme activities (Fig. 4).

Furthermore, the BIOENV procedure identified the C:N ratio, the C:P ratio and MC as the chemical variables that were most strongly correlated with microbial community structure in the LF layer (*ρ* = 0.730). In contrast, pH and lignin concentrations were most strongly correlated with microbial community structure in the H layer (*ρ* = 0.360) (Table 4).

**Table 4:** Combinations of soil variables giving the rank correlations (*ρ*) between chemical properties and microbial PLFA similarity matrices estimated by the BIOENV procedure (n = 45).

LF layer		H layer	
*k*	Best variable combinations (*ρ*)	*k*	Best variable combinations (*ρ*)
1	C:N (0.677)	1	Lig (0.305)
2	C:N, MC (0.711)	**2**	**pH, Lig (0.360)**
3	**C:N, C:P, MC (0.730)**	3	TN, pH, Lig (0.359)
4	SOC, C:N, C:P, MC (0.698)	4	TN, MC, pH, Lig (0.331)
5	SOC, C:N, C:P, MC, Lig (0.680)	5	TN, MC, pH, Cel, Lig (0.307)
6	SOC, C:N, C:P, N:P, MC, Lig (0.664)	6	TN, N:P, MC, pH, Cel, Lig (0.270)

*k* indicates the number of soil variables. Bold type indicates the best combination overall. A total of 10 chemical variables (SOC, TN, TP, C:N, C:P, N:P, MC, pH, Lig and Cel) were included in the analysis. SOC, soil organic carbon; TN, total nitrogen; TP, total phosphorus; MC, moisture content; Cel, cellulose concentration; Lig, lignin concentration

**Figure 5:**
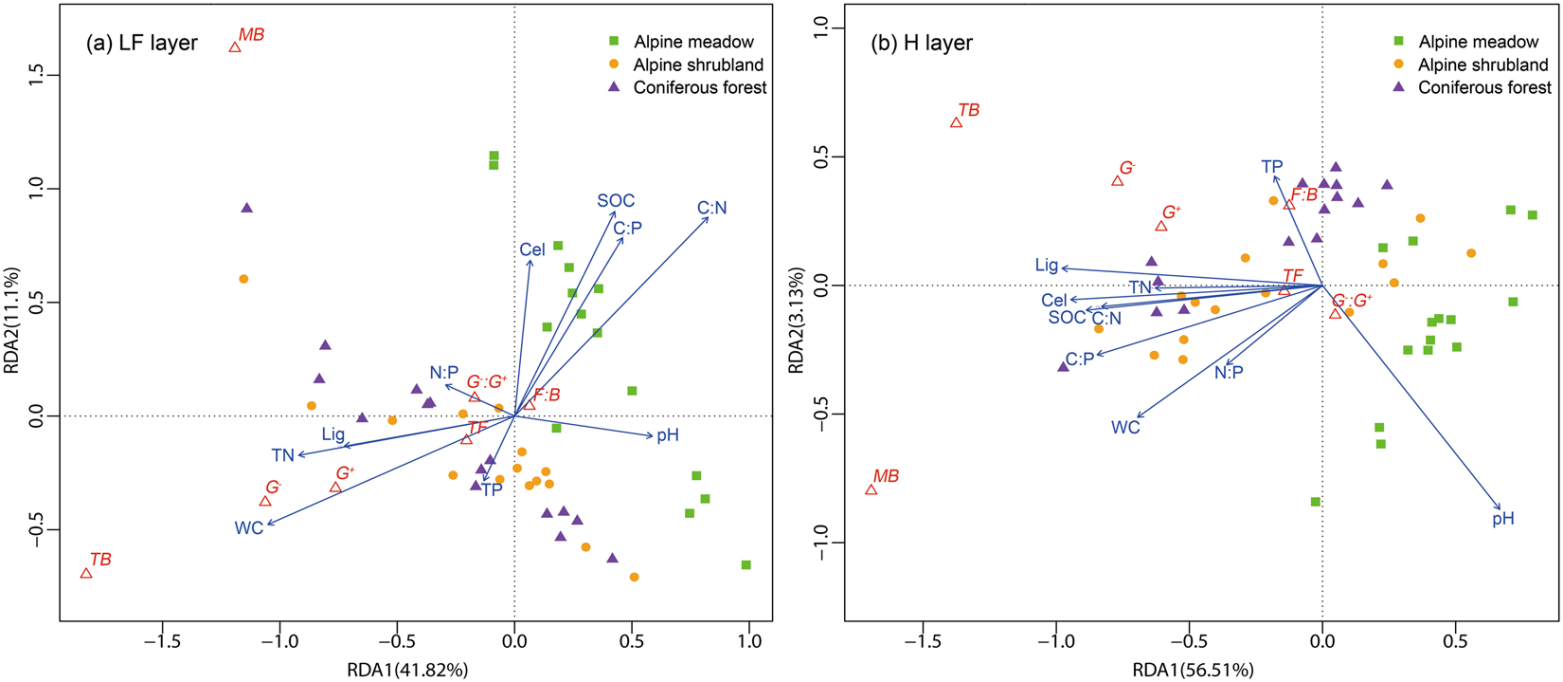
Redundancy analysis (RDA) of microbial indices (red hollow triangles) or litter physicochemical properties (blue lines) with the ordination scores of seven biomarker classes in the LF (**a**) and H (**b**) layers. The microbial indices are total bacteria (TB), total fungi (TF), gram-positive bacteria (G^+^), gram-negative bacteria (G^−^), fungi to bacteria ratio (F:B), ratio of gram-negative bacteria to gram-positive bacteria (G^−^:G^+^) and PLFA microbial biomass (MB). SOC, soil organic carbon; TN, total nitrogen; TP, total phosphorus; MC, moisture content; Cel, cellulose concentration; and Lig, lignin concentration.

The redundancy analysis (RDA) showed that in the LF layer, the first two axes captured 52.72% of the variability of the microbial community structure, whereas RDA1 (the x-axis) and RDA2 (the y-axis) accounted for 41.62% and 11.1% of the variation, respectively (Fig. 5a). SOC, TN, C:N ratio, C:P ratio, pH, MC, cellulose concentration and lignin concentration contributed most to the separation of the samples (pH and cellulose concentration, *p* < 0.05; lignin concentration, *p* < 0.01; others, *p* < 0.001) (Fig. 5a and Table S1). In the H layer, the explanatory variables of litter properties and enzyme activity accounted for 59.64% of the variability in microbial community structure, with 56.51% and 3.13% of the variation explained by RDA1 and RDA2, respectively (Fig. 5b). As observed in the LF layer, SOC, TN, C:N ratio, C:P ratio, MC, cellulose concentration and lignin concentration contributed to the separation of the samples in the H layer (except TN and WC, *p* < 0.01; all *p* < 0.001) (Fig. 5b and Table S1).

In the LF layer, the SOC and C:N and C:P ratios were negatively correlated with the bacterial indices (TB, G^+^ and G^−^) and fungi indices, whereas MC and TN were positively correlated with these microbial indices (Fig. 5a). In the H layer, lignin concentration was positively correlated with the bacterial indices (TB, G^+^ and G^−^) and fungi indices, whereas pH was negatively correlated these microbial indices (Fig. 5b).

## Discussion

### Distribution of lignocellulolytic enzyme activity and microbial community structure

We found that cellulolytic (LiP and MnP) and ligninolytic (EG and BG) enzymatic activities in the LF layer were significantly higher than those in the H layer for all three vegetation types (Fig. 1). Different litter layers (fresh litter layer (L), fermentation layer (F) and humus layer (H)) can represent different stages of litter decomposition^1–3^. Our results clearly indicated that the activities of ligninolytic and cellulolytic enzymes decreased in the later stage of litter decomposition, similar with Snajdr *et al*. (2008), Papa, S., *et al*. (2012) and Fujii *et al*. (2013)^1,18,19^. We argue that litter nutrient content and C:N:P stoichiometry affect the structure and activity of the decomposer community^20,21^. In this study, the C:N, C:P, and N:P ratios; SOC; and lignin and cellulose concentrations were higher in the early stage of decomposition than in the later stage in the three vegetation types (Table 1), which may have stimulated higher enzymatic activities. The proportion of C released in the initial stage of litter decomposition is generally greater than that of N and P, and this stage tends to have higher C availability^22^. However, the C:N and C:P ratios decreased in the later stage of decomposition in the three vegetation types (Table 1); these decreases in the C:N and C:P ratios might have contributed to microbial carbon limitation, indicating that microbial organisms allocated fewer carbon to the production of enzymes that degrade organic matter into monosaccharides. In line with these explanations and our enzyme findings, we did observed that the biomass of microbial PLFAs (TB, TF, G^+^, G^−^ and MB, Fig. 2) decreased over the process of litter decomposition. The significant differences in microbial community structure between the LF and H layers (Fig. 3) were mainly due to the differences in resource availability and the survival strategies of the microorganisms (microbial growth, metabolism, and enzyme production) between the different decomposition stages^7,23^. The correlation analysis revealed positive relationships between enzymatic activities and litter chemical properties (SOC and the C:N, C:P and N:P ratios) (Table 2), confirming the close association between litter substrate quality and enzymatic activity.

On a spatial scale, in general, along with the amount of total soil carbon and its readily utilizable forms, lignocellulose-degrading enzymes (and other enzymes) and microbial biomass were higher in the upper litter layers than in the humus and mineral soil layers^18^. The alpine treeline ecotone from coniferous forests to shrublands and alpine meadows can give rise to dramatic variations in snow depth, snowmelt time, soil temperature, soil moisture and nutrient availability, which, in combination, affect litter decomposition processes^24,25^. Our results showed that significant differences in lignocellulolytic enzymes and microbial characteristics between the two litter layers in the three vegetation types were similar, which could be mainly attributable to the similarity in differences in the litter characteristics (e.g., SOC and the C:N and C:P ratios) across the alpine treeline ecotone (Table 1). This vertical distribution of litter layers was reported in several forest soils ranging from the Mediterranean evergreen forests to temperate forests to boreal ecosystems^1,18,19,26,27^. However, in some temperate and tropical forests, Fujii *et al*. (2013) observed that the activities of lignin peroxidase (LiP) in the H layer were higher than in the LF layer^1^, which may be caused by the highly acidic H layers in that research area. The distribution of the specific basidiomycete fungi and LiP correlated with the high fungal activity in acidic and lignin-rich conditions^28,29^. In this study, the higher activities of LiP were in accordance with the higher lignin contents and higher fungal biomass in the LF layer (Figs 1 and 2 and Table 1).

### Factors affecting microbial community structure

The BIOENV procedure indicated that the variation in microbial community structure during decomposition was mainly affected by the C:N:P stoichiometry, moisture content, pH and lignin concentration. The C:N ratio, C:P ratio and moisture content were the most important determinants of microbial community structure in the LF layer, whereas pH and lignin concentration were the major factors impacting the H layer. It is widely accepted that resource availability can contribute to microbial growth, and there is a popular notion that the C:N:P stoichiometry is strongly related to microbial community structure^30–33^. As discussed before, SOC and available C are more abundant in the LF layer than in the H layer. Available nutrients of low molecular weight are readily lost from the litter through dissolution and leaching in the LF layer^7^. Therefore, microbes prioritize immobilizing the available N and P in the LF layer^34^. This is consistent with our findings that TN, TP and moisture content were positively correlated with most of the bacterial and fungal indices in the LF layer (Fig. 5a). Taken together, these findings suggest that less energy (carbon)-limited and more nutrient (nitrogen or phosphorus)-conserving microbes might be responsible for the negative correlations of SOC, the C:N ratio and the C:P ratio with the bacterial and fungal indices in the LF layer (Fig. 5a). These phenomena can explain why the C:N ratio, C:P ratio and moisture content were the most important determinants of microbial community structure during the initial stage of litter decomposition. These results are consistent with our previous findings that in the early decomposition stage, the activities of β-1,4-exoglucanase and β-1,4-glucosidase appeared to be limited by the N and P contents of the substrate, whereas in the late decomposition stage, the activities of β-1,4-endoglucanase and β-1,4-glucosidase were mainly limited by the C and N contents^5^.

However, at later stages of decomposition, microorganisms allocate more resources to the decomposition of recalcitrant substances, such as lignin, thereby facilitating their growth and metabolism in the organic layer^35,36^. This mainly occurs because at an early stage of decomposition, water-soluble substances and the unshielded hemicellulose/cellulose content decrease quickly relative to the decrease in lignin due to microbial decomposition and utilization^36^. Furthermore, under conditions of low nutrient availability, microbial activity is more easily affected by abiotic factors^7,37^. A more neutral soil pH can enhance biological N fixation by increasing the availability of nutrients^38^. However, in the present study, we observed a relatively low pH in the H layer, particularly in coniferous forest (Table 1). Consistent with these observations, we observed that lignin concentration and pH had major influences on the microbial community structure and close associations with microbial indices in the H layer (Fig. 5b and Table 4).

## Conclusions

Our results indicated that the activities of ligninolytic enzymes (LiP and MnP) and cellulolytic enzymes (EG and BG) as well as the biomass of microbial PLFAs (TB, TF, G^+^, G^−^ and MB) were higher during the initial stage of litter decomposition in the three vegetation types (coniferous forest, alpine shrubland and alpine meadow). PERMANOVA demonstrated that the soil microbial community varied significantly over the course of litter decomposition in the three vegetation types. VPA demonstrated that the variation in the composition of the microbial community in the litter horizon was well explained by physicochemical properties (e.g., soil organic carbon, total nitrogen and total phosphorous) rather than by enzymatic activities. Additionally, the BIOENV procedure showed that the C:N ratio, C:P ratio and moisture content were the most important determinants of microbial community structure during the initial stage of litter decomposition, whereas pH and lignin concentration were the major factors influencing microbial community structure during the later stage. The results of RDA and BIOENV revealed that in the initial stage of litter decomposition with higher available nutrients (particularly available carbon), the microbial indices were negatively correlated with SOC, the C:N ratio and the C:P ratio, suggesting that microbes prioritize immobilizing the available N and P. In contrast, in the later stages with relatively scarce nutrients, microbes adopt strategies to utilize recalcitrant substances such as lignin and are more sensitive to non-nutrient factors, such as pH. These findings indicate that litter quality drives the differentiation of microbial communities in the litter horizon across an alpine treeline ecotone in the eastern Tibetan Plateau.

## Materials and Methods

### Study site and sample collection

Soil samples were collected in an alpine treeline ecotone at the Long-term Research Station of Alpine Forest Ecosystems (Zhegu Mountain region), an important riparian zone located at the eastern edge of the Tibetan Plateau in Sichuan Province, China (31°51′428′′N, 102°41′230′′E). This alpine treeline ecotone offers a natural experimental platform for studying changes in the characteristics of the microbial community during different stages of decomposition in the context of landscape-scale geologic, vegetation and climate gradients. This area contains mixed coniferous forest, broad-leaved forest, dark coniferous forest, alpine shrubland, alpine meadow from valley to hilltop, and alpine desert above 4500 m above sea level (a.s.l.); the timberline occurs at approximately 4000 m a.s.l. and is well preserved. The mountainous region is characterized by steep terrain and is highly dissected by slopes > 35°. The annual average temperature ranges from 6 to 12 °C, with average temperatures of −8 °C and 12.6 °C in January and July, respectively. The annual precipitation ranges from 600 to 1100 mm, and the annual evaporation ranges from 1000 to 1900 mm^17^. The dominant plant species in the coniferous forest are Minjiang fir (*Abies faxoniana*) and alpine rhododendron (*Rhododendron taliense*). The dominant shrub species in the coniferous are *Rhododendron taliense*, *Rhododendron wiltonii*, and *Lonicera myrtillus*. The dominant shrub species in the alpine shrubland are *Salix paraplesia*, *Sorbus rufopilosa*, *Lonicera lanceolata*, *Rosa omeiensis*, and *Berberis silva-taroucana*. There are clear transitions of herbaceous species from the coniferous forest to alpine shrubland and meadow. The dominant herbaceous species in the coniferous forest are *Kobresia macrantha*, *Cystopteris moupinensis*, *Senecio winklerianus*, and *Ligularia sagitta*. The dominant herbaceous species in the alpine shrubland include *Epilobium angustifolium*, *Deyeuxia scabrescens*, and *Gentiana scabra*. The alpine meadow is dominated by *Ajuga ovalifolia*, *Festuca wallichanica*, *Polygonum paleaceum*, and *Pedicularis roylei*. Based on United States Department of Agriculture Soil Taxonomy, the soils in the coniferous forest and shrubland are classified as Cryumbreps, and the soil type of the alpine meadow is Histosols^17^.

Three 50 × 200 m transects were placed along the contour (with the same mountain aspect and similar slope) in the coniferous forest (3900 to 3950 m a.s.l.), alpine shrubland (4000 to 4050 m a.s.l.), and alpine meadow (4200 to 4250 m a.s.l.). Fifteen randomly selected sample plots (plot size 2 × 2 m), each separated by more than 5 m, were placed along each transect. The LF layer is the upper 2 cm layer that contains easily recognizable plant remains that show some discoloration^4,6^. The 2 to 5 cm layer consists of humified material without recognizable plant structures except for stems and coarse root remains, and it is referred to as the H layer^4^. A total of 90 litter samples (3 transects × 2 layers × 15 plots, approximately 200 g of each sample) were collected in October 2014. The samples were immediately refrigerated at 4 °C and shipped on ice to the laboratory, where they were screened for impurities and homogenized. Samples from the LF layer were cut into approximately 0.25 cm^2^ pieces, whereas the samples from the H layer were sieved using a 2 mm sieve. Samples from each sample plot were divided into three subsamples: the first was frozen at −20 °C for subsequent enzymatic activity analysis; the second was frozen at −70 °C for PLFA analysis; and the third was air-dried for chemical analysis.

### Soil chemical analyses

All of the samples from the LF and H layers of each transect were characterized regarding the following physicochemical properties. Moisture content (MC) was assessed using the conventional oven-drying and weighing method (105 °C for 24 h), and pH was measured using a milled litter to solution (water) ratio of 1:2.5 (w/v). The SOC content was determined using the dichromate oxidation ferrous sulfate titration method, and the TN and TP contents were determined by the Kjeldahl method and molybdenumblue colorimetry, respectively; lignin and cellulose contents were measured using the acid detergent lignin method^39^. The stoichiometric ratios (C:N, C:P and N:P) were calculated using SOC, TN and TP.

### Enzyme extraction and assays

Ligninolytic and cellulolytic enzymatic activities that involve LiP, MnP, EG and BG were measured according to the methods of Criquet *et al*.^40–43^, with minor modifications. First, 4 to 9 g of freshly powdered litter (<0.5 mm) was extracted overnight in 15 mL of a 0.1 M CaCl_2_ solution containing 0.05% Tween 80 and 0.40 g of polyvinylpolypyrrolidone at 4 °C, and the suspension was centrifuged at 12000 g for 20 min at 4 °C. The supernatant was subsequently dialyzed for 48 h at 4 °C in 14-kDa molecular mass cut-off cellulose dialysis tubing against a frequently exchanged 2 mM bis-tris (bis [2^-^hydroxyethyl] imino-tris [hydroxymethyl] methane) buffer, pH 6.0. The extracts were boiled for 15 min to serve as controls for enzymatic activity; a reaction mixture without Mn served as a control for MnP activity. Unless otherwise indicated, all enzymatic activities were analyzed at optimal pH values (3.0, 4.5, 6.0 and 5.0 for LiP, MnP, EG and BG, respectively) and optimal temperature (Table 5). The enzyme commission (EC) number and specific substrates are provided in Table 5. One unit of enzymatic activity was defined as the amount of enzyme needed to form 1 nmol min^−1^ of reaction product and expressed as U g^−1^ dry matter {(DM = wet litter mass × (oven dry mass/wet litter mass)} (nmol min^−1^ g^−1^ DM).

**Table 5:** Extracellular enzymes assayed in leaf litter, with abbreviations used in this study, enzyme commission numbers (EC), and corresponding substrates.

Enzyme	Abbreviation	EC	Substrate	Incubation time and temperature
Manganese peroxidase	MnP	EC 1.11.1.13	Phenol red	5 min at 30 °C
Lignin peroxidase	LiP	EC 1.11.1.14	Azure B	5 min at 30 °C
Endo-1-4-β-glucanase	EG	EC 3.2.1.4	Carboxymethylcellulose	1 h at 50 °C
1,4-β-glucosidase	BG	EC 3.2.1.21	*p*-nitrophenyl-β-D-glucoside	40 min at 40 °C

### Phospholipid fatty acid analysis

We investigated the soil PLFA composition to evaluate the changes in the microbial biomass with litter layer among three vegetation types and as an index of the viability of the structure of the microbial community^20^. Phospholipid fatty acids were extracted, fractionated and methylated as described by Bossio and Scow^44^, with slight modifications. A total of 1 g of fresh subsample was extracted using a mixture of chloroform, methanol and potassium phosphate buffer (1:2:0.8 v/v/v). The phospholipids in the concentrated extracts were separated on a silica gel column by sequential elution with organic solvents of increasing polarity and then saponified and methylated to form fatty acid methyl esters (FAMEs). Individual FAMEs were identified by gas chromatography/mass spectrometry (GC/MS, Model QP-2010, Shimadzu, Japan). Peak areas were converted to nanomoles per gram of dry soil (nmol g^−1^ DM) using internal standards (19:0 nonadecanoic methyl ester).

The following PLFAs were used as markers for specific groups: total bacteria (TB): i15:0, a15:0, 16:1ω7c, i17:0, a17:0, cy17:0 and cy19:0; total fungi (TF): 18:1ω9c and 18:2ω6c; gram-positive (G^+^) bacteria: i15:0, a15:0, i17:0 and a17:0; and gram-negative (G^−^) bacteria: 16:1ω7c, cy17:0 and cy19:0^44–47^. The sum of all PLFAs described above together with the unspecific PLFAs (15:0, 16:0, 16:1ω5t, 17:0 and 18:0) were used to define the microbial community composition and to indicate the microbial biomass, and the ratios of fungi to bacteria (F:B) and G^−^:G^+^ were calculated^48,49^.

### Statistical analyses

We used a Mann-Whitney U test to determine the differences in litter physicochemical properties, enzymatic activity and microbial PLFA between the two litter decomposition stages for the three vegetation types. A Pearson correlation analysis was used to evaluate the relationships between litter physicochemical properties and enzymatic activity. The Mann-Whitney U test and Pearson correlation analysis were performed using SPSS Statistics for Windows, version 20.0 (IBM Corp., Armonk, NY, USA). To visually interpret community dissimilarity, non-metric multidimensional scaling ordination (NMDS) was conducted, and PERMANOVA was performed to test whether a significant difference in bacterial community composition was present between the two litter decomposition stages in each of the three vegetation types. Both NMDS and PERMANOVA were performed based on Bray–Curtis dissimilarity (distance) of variability of all PLFA biomarkers in the samples. The redundancy analysis (RDA) (length of gradient < 3 for microbial community variables) and the BIOENV procedure (with microbial communities calculated using Bray-Curtis dissimilarity and litter chemical properties calculated using Euclidean distance) was used to identify the response of the microbial community to environmental variation. The significance of the PERMANOVA and the RDA results was tested with a Monte Carlo permutation test (*permutations* = 999). Microbial community structure data were analyzed in R version 3.3.2 (using the *vegan* package; R Development Core Team, 2011).

## Electronic supplementary material

Supplementary Table S1
