# Supplementary material for: Litter quality drives the differentiation of microbial communities in the litter horizon across an alpine treeline ecotone in the eastern Tibetan Plateau
Source: Sci Rep. 2018 Jul 3;8:10029. doi: 10.1038/s41598-018-28150-1 (PMC6030219; doi:10.1038/s41598-018-28150-1)
Supplement: Supplementary file 1 — Supplementary Table S1 [file 41598_2018_28150_MOESM1_ESM.doc]

**SUPPLEMENTARY INFORMATION TO**

**Litter quality drives the differentiation of microbial communities in the litter horizon across an alpine treeline ecotone in the eastern Tibetan Plateau**

Authors: Haifeng Zheng^&a^, Yamei Chen^& a^, Yang Liu* ^a^, Jian Zhang ^a^, Wanqing Yang ^a^, Lin Yang ^a^, Hongjie Li ^a^, Lifeng Wang ^a^ Fuzhong Wu ^a^, Li Guo^b^

**First author’s Orcid ID:** 0000-0002-1999-1276

Address:

1. *Key Laboratory of Ecological Forestry Engineering of Sichuan Province, Institute of Ecology and Forestry, Sichuan Agricultural University, Chengdu 611130, China*
2. *College of Landscape Architecture, Sichuan Agricultural University, Chengdu 611130, China*

***Corresponding Author:** E-mail address: [sicauliuyang@163.com](mailto:sicauliuyang@163.com); telephone: +8618683506810

^&^These authors contributed equally to this work.

**Supplementary Information**

**Supplementary Table S1** Explanatory value of single physicochemical variables for the microbial community based on Monte Carlo tests conducted with 999 permutations (n = 45).

| Variable | LF layer (*p* < 0.001) | | | H layer (*p* < 0.001) | | |
| --- | --- | --- | --- | --- | --- | --- |
|  | RDA1 | RDA2 | *R*^2^ | RDA1 | RDA2 | *R*^2^ |
| SOC | 0.743 | 0.670 | 0.388 *** | -0.998 | 0.070 | 0.416*** |
| TN | -0.934 | -0.358 | 0.430 *** | -0.993 | 0.122 | 0.212 ** |
| TP | -0.738 | -0.674 | 0.038 | -0.660 | 0.751 | 0.080 |
| C:N | 0.829 | 0.560 | 0.690 *** | -0.997 | 0.076 | 0.365 *** |
| C:P | 0.771 | 0.637 | 0.346 *** | -0.999 | -0.052 | 0.377 *** |
| N:P | -0.999 | -0.047 | 0.033 | -0.931 | -0.364 | 0.082 |
| MC | -0.899 | -0.438 | 0.688 *** | -0.955 | -0.296 | 0.288 ** |
| pH | 0.975 | 0.221 | 0.143 * | 0.796 | -0.605 | 0.546 *** |
| Cellulose | 0.562 | 0.827 | 0.132 * | -0.995 | 0.100 | 0.477 *** |
| Lignin | -0.934 | -0.356 | 0.267 ** | -0.985 | 0.171 | 0.528 *** |

SOC, soil organic carbon; TN, total nitrogen; TP, total phosphorus; MC, moisture content. “*”, “**”, and “***” indicate significance at the 0.05, 0.01, and 0.001 levels, respectively.
